# The dog and rat olfactory receptor repertoires

**DOI:** 10.1186/gb-2005-6-10-r83

**Published:** 2005-09-28

**Authors:** Pascale Quignon, Mathieu Giraud, Maud Rimbault, Patricia Lavigne, Sandrine Tacher, Emmanuelle Morin, Elodie Retout, Anne-Sophie Valin, Kerstin Lindblad-Toh, Jacques Nicolas, Francis Galibert

**Affiliations:** 1 UMR 6061, Génétique et Développement CNRS-Université de Rennes 1, 35043 Rennes Cedex, France; 2 IRISA, campus de Beaulieu, 35042 Rennes Cedex, France; 3 Broad Institute of MIT and Harvard, Charles Street, Cambridge, MA 02141, USA; 4 NIH/NHGRI/50 South Drive, MSC 8000, Bethesda, MD 20892-8000, USA

## Abstract

**Background:**

Dogs and rats have a highly developed capability to detect and identify odorant molecules, even at minute concentrations. Previous analyses have shown that the olfactory receptors (ORs) that specifically bind odorant molecules are encoded by the largest gene family sequenced in mammals so far.

**Results:**

We identified five amino acid patterns characteristic of ORs in the recently sequenced boxer dog and brown Norway rat genomes. Using these patterns, we retrieved 1,094 dog genes and 1,493 rat genes from these shotgun sequences. The retrieved sequences constitute the olfactory receptor repertoires of these two animals. Subsets of 20.3% (for the dog) and 19.5% (for the rat) of these genes were annotated as pseudogenes as they had one or several mutations interrupting their open reading frames. We performed phylogenetic studies and organized these two repertoires into classes, families and subfamilies.

**Conclusion:**

We have established a complete or almost complete list of OR genes in the dog and the rat and have compared the sequences of these genes within and between the two species. Our results provide insight into the evolutionary development of these genes and the local amplifications that have led to the specific amplification of many subfamilies. We have also compared the human and rat ORs with the human and mouse OR repertoires.

## Abstract 2

An almost complete list of odorant receptor genes in the dog (1,094 genes) and the rat (1,493 genes) is described. A comparison of odorant receptor repertoires in rat, dog, mouse and human is also included.

## Background

Olfaction is one of the senses developed by animals during the course of evolution for communication with the external world, making it possible to identify prey and to avoid danger. The detection of volatile odorant molecules is a complicated process, the first step of which involves specific binding to specialized receptors. Olfactory receptors (ORs) - encoded by the largest known gene superfamily in the mammalian genome, also known as the olfactory subgenome [1] - are expressed on the surface of the cilia of the olfactory sensory neurons lining the neuroepithelium in the nasal cavity. OR proteins belong to the G protein-coupled receptor superfamily, which is characterized by the presence of seven hydrophobic transmembrane domains. G-protein coupling facilitates the transduction of a signal from the activated olfactory sensory neurons to olfactory glomeruli on the anterior surface of the brain. Secondary neurons then convey the signal to the upper part of the brain for further processing and identification of the odorant molecule. Each OR can recognize several chemically related molecules, and a specific odorant may bind to several ORs [2]. This combinatorial coding system has been only partly deciphered, with only about 20 or so ligand-receptor pairs of the thousands possible decoded [2-13]. OR genes were first recognized by Buck and Axel [14] and recent genome sequence data mining has led to the identification and characterization of about 650 to 900 genes in humans [15,16] and 1,200 to 1,500 genes in mice [17-19]. The olfactory repertoire of rat has been estimated to contain 1,700 to 2,000 genes [20], whereas that of the dog has been estimated at 1,300 genes [21,22].

We report here a more thorough inventory of the dog and rat repertoires and a comparison between them. We also compare the sequences and genome organization of these two repertoires and of the human and mouse repertoires, and provide evidence for the evolution of OR repertoires by local duplications leading to the independent expansion of some subfamilies. This evolutionary process accounts for differences between the OR repertoires.

## Results

### The dog OR gene repertoire

We searched the 35.9 million sequencing reads of the 7.5 × shotgun sequence [23] for five amino acid patterns characteristic of the dog OR and retrieved almost 60 thousand reads, corresponding to a total of 40,408,752 nucleotides. We checked the quality of each sequence read and trimmed both extremities before assembly with Cap 3 software [24]. Sequences were assembled with great care, using dedicated parameter settings to prevent the assembly of reads corresponding to different genes. A threshold of 97% identity over 25 nucleotides was the lowest limit at which a maximum of false assemblies could be eliminated without too great a loss of assembly power. With this setting, we obtained 6,727 contigs, within which we looked for the five patterns in defined positions characteristic of the OR family. We finally identified 1,058 unique consensus sequences as OR genes.

We also independently searched CanFam1.0 [25] with the same five amino acid patterns and retrieved 1,014 OR genes. We compared these two sets of genes and found that 1,003 OR genes were identified by both approaches, with 55 identified by partial genome assembly only and 11 identified by whole genome assembly only. These differences probably reflect assembly problems that have not yet been solved, requiring *in vitro *cloning experiments to obtain precise knowledge of the dog OR repertoire. We compared this set of genes with the 661 genes previously characterized [21] and identified 25 genes present only in the 661 gene pool, possibly reflecting the fact that a 7.5 × shotgun sequence covers about 98% of the genome [26]. The lowest current estimate for the size of the canine OR repertoire is, therefore, 1,094 genes (1,003 + 55 + 11 + 25). We identified 27 additional sequences corresponding, at best, to very highly pseudogenized OR genes, which were therefore excluded from subsequent analysis.

### The rat OR gene repertoire

We screened the whole rat genome assembly (release Rnor3.1) [20] and identified 1,493 genes as OR genes on the basis of the order and spacing of the five characteristic amino acid patterns. We also identified about 350 sequences that contained only a subset of the five patterns, dispersed throughout the genome assembly, corresponding to additional genes that might eventually be identified as true OR genes after genome sequencing has been completed. Most of these sequences are unlikely to be true OR gene sequences, however, as they diverge considerably from the consensus. They are classified as pseudogenes in GenBank, but we prefer to reserve the term 'pseudogene' for complete genes with well identified mutations closing the reading frame. We therefore excluded these highly modified sequences from subsequent analysis.

### Genes and pseudogenes

Translation of the dog and rat gene sequences made it possible to identify pseudogenes and to determine the number of mutations closing the open reading frame (ORF). Consistent with earlier observations, 20.3% and 19.5% of dog and rat OR genes, respectively, were identified as pseudogenes. A single frame-closing mutation was detected in 78 of the 222 dog pseudogenes with unambiguously annotated start and stop codons; 43 of the pseudogenes had 2 such mutations, and 101 had 3. Similar results were obtained for the rat, with 153 pseudogenes having a single mutation, 48 having two mutations and 91 having three or more mutations closing the reading frame. Pseudogenes with more than one mutation closing the ORF are certainly real pseudogenes. Not all pseudogenes with a single frame-closing mutation are real pseudogenes, however, as shown by sequence polymorphism analysis [27].

### Dog and rat OR gene location

We mapped 562 of the 661 dog genes identified by *in vitro *and *in silico *cloning [21] on the radiation hybrid panel. Their distribution closely resembled that of their human counterparts, taking into account the greater fragmentation of the dog karyotype, with its 38 autosomes in addition to the X and Y sex chromosomes [21]. The precise location of 902 of the 1,094 OR genes identified in this study was given in CanFam1.0 and 61 of these genes have been attributed to a given *Canis familiaris *chromosome by radiation hybrid mapping only, with 131 remaining unassigned.

We noted no conflict between previous radiation hybrid map positions and those deduced from CanFam1.0. The newly mapped OR genes did not affect the general picture; they simply increased the size of the known clusters. The only real change observed concerned *C. familiaris *chromosome 2, which was previously considered devoid of OR genes but has now been assigned a small cluster of two genes and two pseudogenes. Finally, pseudogenes were found in almost all clusters (Additional data file 1).

Similar results were obtained concerning the distribution of the 1,493 genes and pseudogenes identified in the rat genome (Additional data file 2). Comparison of the four known mammalian OR repertoires (human, mouse, rat and dog) showed that regardless of differences in karyotype and repertoire size, OR genes were distributed in very similar numbers of clusters, as defined by groups of OR separated by more than one megabase (Table 1).

### Amino-acid sequence comparison

**Figure 1:**
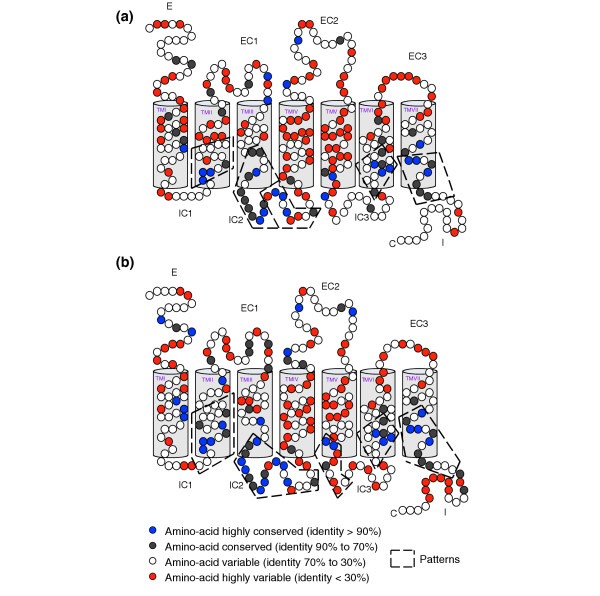
Positions of conserved and variable amino acids in 1,009 dog and 1,470 rat OR proteins. **(a) **Comparison of 1,009 dog OR genes. **(b) **Comparison of 1,470 rat OR genes. E and EC, extracellular domain; I and IC, intracellular domain; TM, transmembrane domain.

We aligned all the dog and rat OR amino acid sequences to determine the level of variability at each amino acid position. Figure 1 shows schematic diagrams of OR proteins, with a color scheme used to indicate the level of identity.

With the exception of the amino-terminal position, no amino acid position is entirely invariant. The dog repertoire was smaller than that of the rat, and contained fewer highly conserved (≥90%) positions: 23 in dog OR proteins versus 31 in rat OR proteins. This lower level of conservation and the larger number of subfamilies identified in the dog repertoire indicate that the dog has a more diverse repertoire than the rat.

Twenty of these highly conserved positions are common and correspond to the same amino acid in both repertoires. Furthermore, 15 positions in dog sequences and 21 in rat sequences correspond to the amino acid identified in PRATT patterns [28]. Transmembrane domains IV and V have the highest proportions of highly variable amino acids, consistent with the role of these domains in ligand recognition and binding [29,30].

### Phylogenetic comparison

We then used ClustalW [31] to compare the 1,009 complete amino acid sequences for dog ORs with the 1,493 complete amino acid sequences for rat ORs and constructed two independent trees. Based on previously used thresholds (40% and 60% amino acid identity for distinguishing families and subfamilies, respectively), a similar pattern of organization to that reported for the human [32] and mice [19] repertoires was observed (Table 2).

The human repertoire is the smallest of the four known mammalian repertoires and consists of the smallest number of families, 17. Like the dog repertoire, however, it can be divided into 300 subfamilies. The rat repertoire contained only 282 subfamilies (Additional data file 3), despite being the largest of the four repertoires. The large number of subfamilies in humans probably reflects the much larger number of pseudogenes, with up to 126 subfamilies consisting entirely of pseudogenes, rather than true diversification of this repertoire. In contrast, the larger number of subfamilies in the dog repertoire reflects a higher level of diversification. Accordingly, the subfamilies that varied considerably in size were smaller in dog than in rat: 1 to 31 genes for the dog and 1 to 61 genes for the rat (Additional data file 3).

Pseudogenes were detected in both classes and in all families and subfamilies, but were unevenly distributed. Class I (193 and 150 genes for dog and rat, respectively) included fewer pseudogenes (17% and 13% for dog and rat, respectively) than class II (23% and 20% pseudogenes for dog and rat, respectively).

Even greater variability was observed in families and subfamilies. For example, in family 6 (class II), 34% of the 134 OR genes in dog and 41% of the 210 OR genes in rat were pseudogenes. In family 10 (class II), 13% of the 46 genes in dog and 20% of the 51 genes in rat were identified as pseudogenes (see also Additional data file 3).

**Figure 2:**
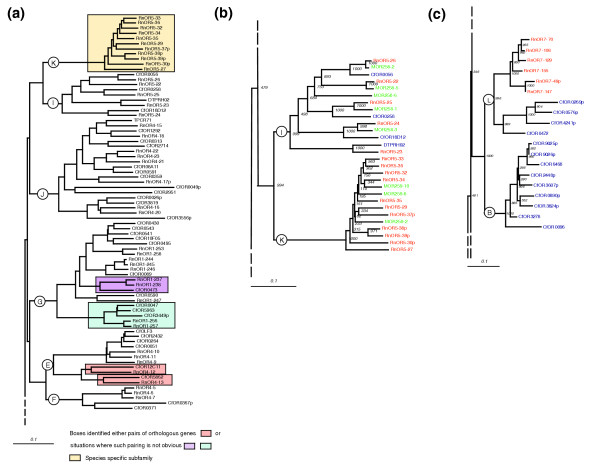
Analysis of OR families by phylogenetic comparison. OR sequences used to construct the phylogenetic trees correspond to the dog and rat sequences retrieved in this work or taken from [15] (human) and [19] (mouse). **(a) **Magnification corresponding to a part of family 2, including subfamilies E to G and I to K (general combined phylogenetic tree as provided in Additional data files 4 to 20). Circled letters identify dog and rat subfamilies. **(b) **Rat, dog and mouse subfamilies 2I and 2K (the corresponding subfamilies do not exist in humans). Rat genes are in red, dog genes in blue and mouse genes in green. **(c) **Subfamilies 6AL and 6B (note that subfamily 6AL is present in rat and dog repertoires but is absent from the human and mouse repertoires and that subfamily 6B is present only in the dog repertoire). The color code is the same as in (b).

Orthologous genes are defined as genes with the same evolutionary background in different species. They are usually very similar in sequence and they are assumed to have similar or identical functions. Orthologous OR genes would, therefore, be expected to bind the same ligand molecule, although this might not always be the case [4]. To facilitate the identification of pairs of orthologous genes in the dog and rat repertoires and of genes belonging to the same families and subfamilies, we constructed a single tree with data from both species (Additional data files 4 to 20). Figure 2a is a magnification of a region of this common tree, corresponding to dog and rat family 2, which belongs to class II and consists of 12 dog and 12 rat subfamilies. The identification of orthologous gene pairs such as RnOR4-13/CfOR5862 and RnOR4-12/CfOR12C11 is straightforward; however, we frequently observed situations in which one dog gene corresponded to two or more rat genes (for example, dog gene CfOR0473 and rat genes RnOR1-237, RnOR1-238), or vice versa. There are even more complex situations in which a small group of OR genes in one species corresponds to a group of genes in the other species. In these cases, it is not possible to pair dog and rat orthologous genes. An example of this situation is provided by the three dog genes CfOR0047, CfOR5963 and CfOR3449, which correspond to the two rat genes, RnOR1-256 and RnOR1-257.

Analysis of the combined tree also identified subfamilies that had expanded in one species but not in the other, or were present in only one of the two species. For example, subfamily 7A contained 31 genes in dog, 11 in rat, 3 in human but none in mouse, and subfamily 2K included 11 genes in rat but was not found in dog. This subfamily was absent in humans but was found in mice, albeit with only three members (Figure 2b). The reverse situation was observed for subfamily 6B, which contained nine genes in dog but was absent from the rat, human and mouse repertoires (Figure 2c). Other examples are provided in Additional data files 4 to 20.

**Figure 3:**
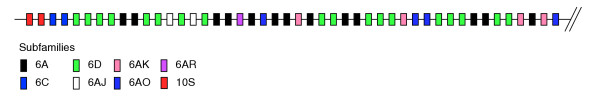
Gene order of families 6 and 10 in cluster Rno7@3-9. This diagram shows the alignment of the first 46 genes of this rat cluster. As shown by the different colors, genes of different subfamilies are intermingled.

It has been shown that OR genes from the same subfamily tend to be clustered [15]. Only 22 dog subfamilies (134 OR genes) and 11 rat subfamilies (168 OR genes), corresponding to only 7% and 4% of all subfamilies, respectively, were found on more than one chromosome. Furthermore, from the way in which rat genes are named, it rapidly became apparent that the order of the genes in the genome tends to respect phylogenetic order, as shown by rat subfamily 2K (Figure 2a), which consists of 11 genes identified by digits 027 to 039. Also rat cluster Rno5@138-139 has two parts, the first containing the five OR genes of subfamily 2I, and the second containing the 11 OR genes of subfamily 2K. The homologous cluster in dog is called 15@3 and contains only four genes belonging to subfamily 2I. One of the rat 2I subfamily members may have undergone several rounds of duplication, leading to the creation of a specific rat 2K subfamily. Rat OR gene 5-26, from subfamily 2I, is the fifth gene in the cluster and has the highest scores for identity to the members of the 2K subfamily. A duplication of this gene may have created the first member of the 2K subfamily in rat, accounting for the existence of a species-specific subfamily within a cluster. In some cases, gene order in the genome does not respect phylogenetic order, as for rat cluster 7@3-9 (Figure 3), which contains a mixture of genes from different subfamilies.

## Discussion

We retrieved 1,493 OR genes from the most recent rat genome sequence assembly (Rnor3.1) and 1,094 OR genes from the 7.5 × dog shotgun sequence (sequencing traces and CanFam1.0). The rat repertoire described here differs in size from that reported in GenBank, mainly because we did not take into account several hundred sequences corresponding to very incomplete genes, with only one to three patterns, probably corresponding to highly disabled pseudogenes.

The identification of a string of nucleotides encoding an OR is straightforward because all ORs are of similar length and have the same general structure, with seven hydrophobic transmembrane domains. They are also characterized by several amino acid patterns and an intron-less coding sequence of 940 ± 30 base pairs. In contrast to the ease with which a single OR can be identified, it is extremely difficult to determine the complete repertoire of OR genes in a given mammalian genome. This is due not only to the large size of the OR repertoire, exceeding 1,000 genes, but also to the high level of variability between OR genes, which display 34% to 99% identity [19]. Any shotgun sequence assembly that does not address this problem specifically is prone to errors, generating contigs with sequencing reads corresponding to very similar paralogous genes. The difficulty in assembling reads correctly is further increased by the fact that many, if not all, genes have two allelic variants that may differ by a large number of Single Nucleotide Polymorphism (SNP) [27]. The difficulties involved in identifying mammalian OR repertoires correctly and thoroughly are illustrated by the different results we obtained by retrieving OR genes from CanFam1.0 and from non-assembled reads. Similar difficulties in identifying a complete OR repertoire are also evident in studies of the mouse repertoire, which has been estimated at 1,500 [18] and 1,200 [19] genes.

We believe that our estimates of the numbers of genes in these two ORs (1,493 rat OR genes and 1,094 dog OR genes) are accurate; however, these estimates are likely to change with time and future sequencing results.

Phylogenetic analyses were used to compare OR amino acid sequences and to organize the repertoire into classes, families and subfamilies. This facilitated the identification of pairs of orthologous genes and, in many cases, groups of paralogous genes in one species orthologous to groups of paralogous genes in the other species. Comparing the results of phylogenetic and syntenic analyses, we found that a series of local duplications had taken place during the evolution of these two genomes, resulting in large repertoires in both species, but with orthologous subfamilies differing in size in the two species and some species-specific subfamilies.

We counted the number of amino acid differences and their frequencies at each position in the OR proteins and found only 23 and 31 positions with a very high level of identity (≥90%) in dog and rat, respectively. Twenty of these positions were common to both species and were occupied by the same amino acid. Conversely, many pairs or small groups of paralogous genes were found to encode proteins displaying up to 99% amino acid identity. As can be seen on the phylogenetic tree, there were fewer subfamilies in rat than in dog and the rat subfamilies were generally larger. Thus, although the rat repertoire is much larger than the dog repertoire, it appears to be less polymorphic. It is unclear to what extent this observation reflects the respective sensing capacities of these two species and the fact that many dog breeds were created to exploit their olfactory function.

## Conclusion

Determination of the sequences of several mammalian genomes has provided an opportunity for counting and comparing the genes comprising these genomes. Such studies have advanced studies of OR gene families, which contain 1,000 to 1,500 different genes, which it would have been impossible to identify by direct cloning and sequencing. We present here complete or almost complete inventories of the dog and rat olfactory repertoires, which we compared by constructing an integrated phylogenetic tree including all the OR sequences. A limited number of OR-ligand pairs have been determined, but there is strong evidence that the products of the OR genes of a given subfamily recognize molecules of similar shape or chemical function. The smaller number of subfamilies identified in the rat repertoire is intriguing and raises questions concerning possible species-specific differences in sensing capacities and the role of dog breeding in enhancing olfactory function.

## Materials and methods

### Pattern discovery

We selected 45 full-length canine OR genes [21] and 200 rat OR genes from already annotated OR genes (GenBank) to define OR-specific patterns with the PRATT program [28] available on the Pattern Discovery Platform [33]. Pattern recognition was based on criteria listed in Table 3. Five patterns distributed along the length of the OR proteins were selected for each species (Table 4).

### OR screening

The unassembled dog 7.5 × sequence, and the 1^st ^assembly release (CanFam1.0) of the dog sequence and the assembled rat genome (Rnor3.1 [20]) were screened with the five patterns identified with PRATT [28]. Screening was initially carried out with STAN (an analyzer based on suffix trees, able to scan genomes for Prosite-type protein patterns, available on the Pattern Matching Platform [33]). We then increased the flexibility of recognition by translating patterns into weighted finite automata [34], allowing arbitrary error thresholds. We scanned for these patterns in all six translation frames, using the Rdisk prototype architecture [35]. The boards of this prototype contain FPGA processors, which were reconfigured to speed up screening by one order of magnitude.

For the dog, the sequences retrieved from the non-assembled sequences were cleaned using quality values from the NCBI web site, as follows: extremities were shortened until the quality value for a window of 10 nucleotides exceeded 20; and sequences with a mean quality value below 15 were eliminated [23]. The resulting processed sequences were assembled with Cap3 software [24] using the following criteria: minimum overlap of 25 nucleotides and identity values of 97% required to prevent illegitimate assembly. A consensus sequence was established for each contig.

### Characterization of OR genes

All retrieved sequences were further analyzed by searching for the five patterns at specific locations. Each consensus OR gene sequence was then translated with the 'Traduction Multiple' program available from the Infobiogen web site [36]. If more than one ORF was possible, as for pseudogenes resulting from insertions or deletions, we used the BlastX program [37,38] to determine the limits of each partial ORF and manually reconstructed the OR protein sequence. The dog and rat OR sequences have been submitted to GenBank and are accessible from the  authors' website [39].

### Classification

Complete OR protein sequences were aligned using ClustalW software [31] and classes, families and subfamilies defined as previously described [40-42]. Trees were constructed with TreeView [43] and the dog ADRB3 gene as the outgroup.

### Genome localization

We localized canine OR genes precisely within the genome by carrying out Blast analysis against CanFam1.0. The coordinates of rat OR genes were taken from the draft genome sequence Rnor3.1.

### OR gene nomenclatures

Canine OR gene sequences are named 'CfORxxxx' for *C. familiaris *olfactory receptor.

The names of the rat OR sequences refer to their chromosomal location, for example, gene RnOR1-061 is the 61st OR gene present on rat chromosome 1, counting from the end of one telomere.

## Additional data files

The following additional data are available with the online version of this paper (and also at the authors' web site [39]). Additional data file 1 is a spreadsheet listing chromosomal locations of dog OR genes and pseudogenes. Additional data file 2 is a spreadsheet listing the chromosomal location of rat OR genes and pseudogenes. Additional data file 3 is a spreadsheet showing the number of rat and dog OR genes and pseudogenes per family and subfamily. Additional data file 4 is a phylogenetic tree for family 2. Additional data file 5 is a phylogenetic tree for family 3. Additional data file 6 is a phylogenetic tree for family 4. Additional data file 7 is a phylogenetic tree for family 5. Additional data file 8 is a phylogenetic tree for family 6. Additional data file 9 is a phylogenetic tree for family 7. Additional data file 10 is a phylogenetic tree for families 8-9. Additional data file 11 is a phylogenetic tree for families 10-19-20-21. Additional data file 12 is a phylogenetic tree for family 12. Additional data file 13 is a phylogenetic tree for family 14. Additional data file 14 is a phylogenetic tree for family 15. Additional data file 15 is a phylogenetic tree for family 16. Additional data file 16 is a phylogenetic tree for families 17-18. Additional data file 17 is a phylogenetic tree for family 51. Additional data file 18 is a phylogenetic tree for family 52. Additional data file 19 is a phylogenetic tree for families 55-57. Additional data file 20 is a phylogenetic tree for family 56.

## Supplementary Material

Additional data file 1 A spreadsheet listing chromosomal locations of dog OR genes and pseudogenes. Clusters are named according to their position, in megabases, from the top of each chromosome. Click here for file

Additional data file 2 A spreadsheet listing the chromosomal location of rat OR genes and pseudogenes. Clusters are named according to their position, in megabases, from the top of each chromosome. Click here for file

Additional data file 3 A spreadsheet showing the number of rat and dog OR genes and pseudogenes per family and subfamily. Families 2 to 21 belong to class II and families 51 to 57, to class I. Click here for file

Additional data file 4 Phylogenetic tree for family 2. Dog and rat OR proteins belonging to the same family were aligned using ClustalW software [31] and a phylogram for each family was constructed using canine ADRB3 gene as the outgroup. Subfamilies are indicated by circled letters. Rat genes are shown in red and dog genes, in blue. Click here for file

Additional data file 5 Phylogenetic tree for family 3. Dog and rat OR proteins belonging to the same family were aligned using ClustalW software [31] and a phylogram for each family was constructed using canine ADRB3 gene as the outgroup. Subfamilies are indicated by circled letters. Rat genes are shown in red and dog genes, in blue. Click here for file

Additional data file 6 Phylogenetic tree for family 4. Dog and rat OR proteins belonging to the same family were aligned using ClustalW software [31] and a phylogram for each family was constructed using canine ADRB3 gene as the outgroup. Subfamilies are indicated by circled letters. Rat genes are shown in red and dog genes, in blue. Click here for file

Additional data file 7 Phylogenetic tree for family 5. Dog and rat OR proteins belonging to the same family were aligned using ClustalW software [31] and a phylogram for each family was constructed using canine ADRB3 gene as the outgroup. Subfamilies are indicated by circled letters. Rat genes are shown in red and dog genes, in blue. Click here for file

Additional data file 8 Phylogenetic tree for family 6. Dog and rat OR proteins belonging to the same family were aligned using ClustalW software [31] and a phylogram for each family was constructed using canine ADRB3 gene as the outgroup. Subfamilies are indicated by circled letters. Rat genes are shown in red and dog genes, in blue. Click here for file

Additional data file 9 Phylogenetic tree for family 7. Dog and rat OR proteins belonging to the same family were aligned using ClustalW software [31] and a phylogram for each family was constructed using canine ADRB3 gene as the outgroup. Subfamilies are indicated by circled letters. Rat genes are shown in red and dog genes, in blue. Click here for file

Additional data file 10 Phylogenetic tree for families 8-9. Dog and rat OR proteins belonging to the same family were aligned using ClustalW software [31] and a phylogram for each family was constructed using canine ADRB3 gene as the outgroup. Subfamilies are indicated by circled letters. Rat genes are shown in red and dog genes, in blue. Click here for file

Additional data file 11 Phylogenetic tree for families 10-19-20-21. Dog and rat OR proteins belonging to the same family were aligned using ClustalW software [31] and a phylogram for each family was constructed using canine ADRB3 gene as the outgroup. Subfamilies are indicated by circled letters. Rat genes are shown in red and dog genes, in blue. Click here for file

Additional data file 12 Phylogenetic tree for family 12. Dog and rat OR proteins belonging to the same family were aligned using ClustalW software [31] and a phylogram for each family was constructed using canine ADRB3 gene as the outgroup. Subfamilies are indicated by circled letters. Rat genes are shown in red and dog genes, in blue. Click here for file

Additional data file 13 Phylogenetic tree for family 14. Dog and rat OR proteins belonging to the same family were aligned using ClustalW software [31] and a phylogram for each family was constructed using canine ADRB3 gene as the outgroup. Subfamilies are indicated by circled letters. Rat genes are shown in red and dog genes, in blue. Click here for file

Additional data file 14 Phylogenetic tree for family 15. Dog and rat OR proteins belonging to the same family were aligned using ClustalW software [31] and a phylogram for each family was constructed using canine ADRB3 gene as the outgroup. Subfamilies are indicated by circled letters. Rat genes are shown in red and dog genes, in blue. Click here for file

Additional data file 15 Phylogenetic tree for family 16. Dog and rat OR proteins belonging to the same family were aligned using ClustalW software [31] and a phylogram for each family was constructed using canine ADRB3 gene as the outgroup. Subfamilies are indicated by circled letters. Rat genes are shown in red and dog genes, in blue. Click here for file

Additional data file 16 Phylogenetic tree for families 17-18. Dog and rat OR proteins belonging to the same family were aligned using ClustalW software [31] and a phylogram for each family was constructed using canine ADRB3 gene as the outgroup. Subfamilies are indicated by circled letters. Rat genes are shown in red and dog genes, in blue. Click here for file

Additional data file 17 Phylogenetic tree for family 51. Dog and rat OR proteins belonging to the same family were aligned using ClustalW software [31] and a phylogram for each family was constructed using canine ADRB3 gene as the outgroup. Subfamilies are indicated by circled letters. Rat genes are shown in red and dog genes, in blue. Click here for file

Additional data file 18 Phylogenetic tree for family 52. Dog and rat OR proteins belonging to the same family were aligned using ClustalW software [31] and a phylogram for each family was constructed using canine ADRB3 gene as the outgroup. Subfamilies are indicated by circled letters. Rat genes are shown in red and dog genes, in blue. Click here for file

Additional data file 19 Phylogenetic tree for families 55-57. Dog and rat OR proteins belonging to the same family were aligned using ClustalW software [31] and a phylogram for each family was constructed using canine ADRB3 gene as the outgroup. Subfamilies are indicated by circled letters. Rat genes are shown in red and dog genes, in blue. Click here for file

Additional data file 20 Phylogenetic tree for family 56. Dog and rat OR proteins belonging to the same family were aligned using ClustalW software [31] and a phylogram for each family was constructed using canine ADRB3 gene as the outgroup. Subfamilies are indicated by circled letters. Rat genes are shown in red and dog genes, in blue. Click here for file

## Figures and Tables

**Table 1:** Distribution of olfactory receptor genes in the four mammalian genomes

	Human*	Mouse^†^	Rat	Dog
Number of loci	51	51	56	49
Number of genes per locus	1-116	1-244	1-265	1-211
Number of loci with only pseudogenes	13	2	8	5

*From [15]. ^†^From [19].

**Table 2:** Distribution of olfactory receptor genes in families and subfamilies

	Number of classes	Number of families	Number of subfamilies
Human*	2	17	300
Mouse^†^	2	Nd	241
Dog	2	23^‡^	300
Rat	2	21	282

*From [32]. ^†^From [19]. ^‡^Note that this number of families is lower than that previously published [21]. This is probably because the published number was calculated from the alignment of the middle part of the sequences, which is more diverse, particularly for transmembrane domains TMIII and TMIV. Nd, not determined.

**Table 3:** Criteria used for pattern recognition with the PRATT program [28]

Parameter	Description	Value
C%	Pattern conservation	95% (dog) 90% (rat)
L	Maximum pattern length	25 amino acids
S	Research complexity	High (E = 0; dog) Medium (E = 1; rat)
PN	Maximum number of pattern symbols	25
PX	Maximum number of consecutive undetermined amino acids	5
FN	Maximum number of flexible gaps	2
FL	Maximum flexibility of a flexible wildcard	3
FP	Maximum flexibility product	10

**Table 4:** Amino acid patterns used to retrieve olfactory receptor genes

Pattern number	Transmembrane domain	Pattern
Dog		
1	TMII	P-M-Y-x-[FL]-L-x(2)-[FL]-[AMS]-x(2)-[DE]
2	TMIII	L-x(1,3)-M-x-[FILY]-D-R-x(2)-A-[IV]-[CS]-x-P-L-x-[HY]-x(3)-[ILM]
3	TMIII	L-x(3)-M-x(0,1)-Y-x-[FLR]-[LY]-x(2)-[FILV]-[ACS]
4	TMVI	K-x-[FL]-[AGHNST]-T-C-x-[AS]-H-x(3)-[AIV]
5	TMVII	N-P-[FILMV]-[IV]-Y-[AGST]-[AILMV]-[KR]-x(2)-[DEKQ]
Rat		
1	TMII	L-[HKNQR]-x-P-M-[FY]-x-[FIL]-L-x(2)-L-x(3)-[DEY]
2	TMIII	M-[AS]-[FLY]-D-R-[FHY]-[AILMV]-A-[IV]-x(2)-P-L-x-[HY]-x(3)-[FILMV]-[DGHKNPRST]
3	TMV	S-Y-x(2)-I-[FILV]-x-[AST]-[FIV]
4	TMVI	K-x-[FILMV]-x-T-C-x-[ACPST]-H-[FILMV]-x(2)-[FILMV]
5	TMVII	P-x-[LMV]-N-P-[FILMV]-x-Y-[ACGST]-x-[KNR]-x-[KNQRT]-[DEKPQ]-[FILMV]
