# Supplementary figures and images for: The dog and rat olfactory receptor repertoires
Source: Genome Biol. 2005 Sep 28;6(10):R83. doi: 10.1186/gb-2005-6-10-r83 (PMC1257466; doi:10.1186/gb-2005-6-10-r83)

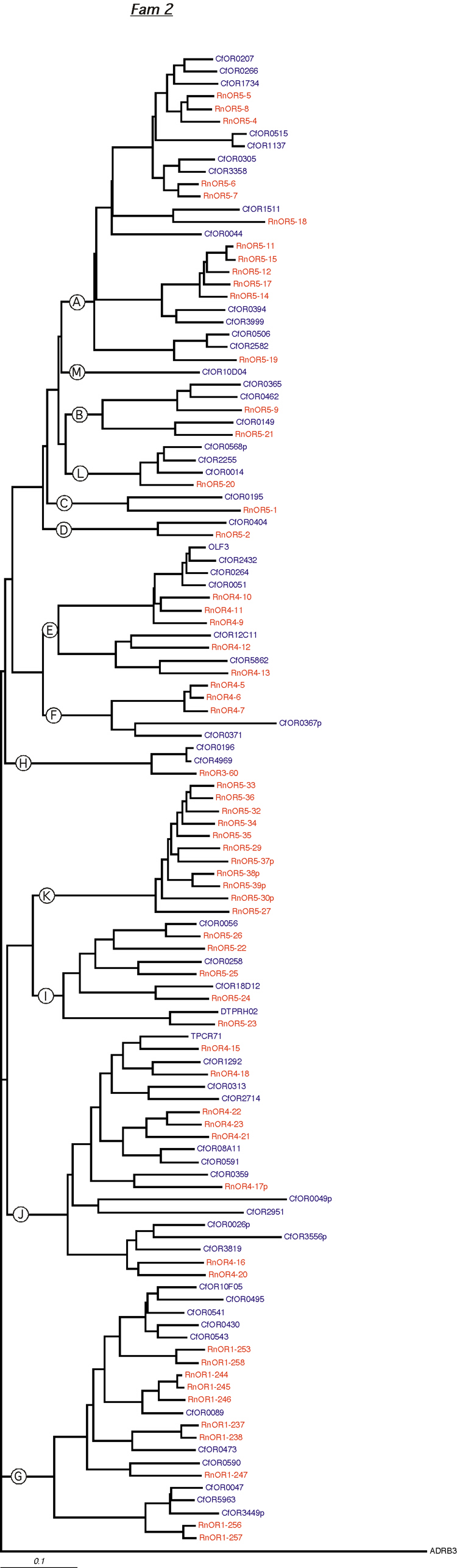

Supplement: Additional data file 4 — Phylogenetic tree for family 2. Dog and rat OR proteins belonging to the same family were aligned using ClustalW software [31] and a phylogram for each family was constructed using canine ADRB3 gene as the outgroup. Subfamilies are indicated by circled letters. Rat genes are shown in red and dog genes, in blue. [file gb-2005-6-10-r83-S4.jpeg]

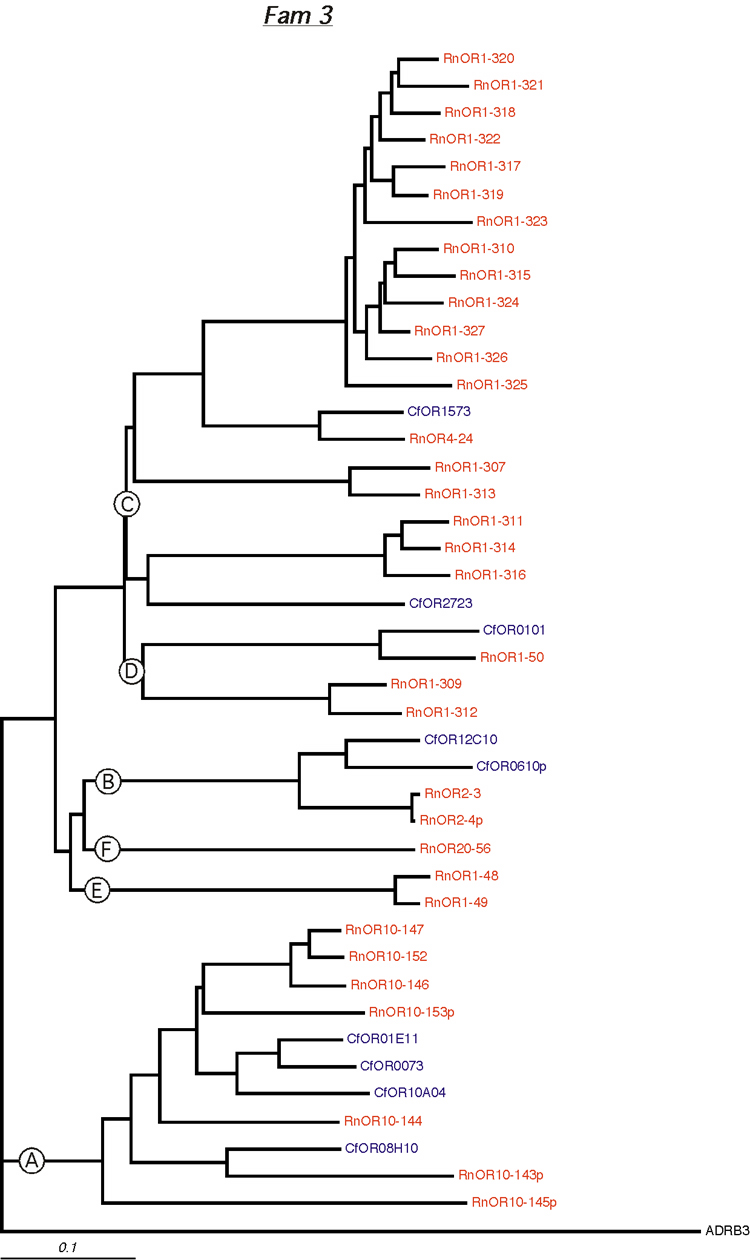

Supplement: Additional data file 5 — Phylogenetic tree for family 3. Dog and rat OR proteins belonging to the same family were aligned using ClustalW software [31] and a phylogram for each family was constructed using canine ADRB3 gene as the outgroup. Subfamilies are indicated by circled letters. Rat genes are shown in red and dog genes, in blue. [file gb-2005-6-10-r83-S5.jpeg]

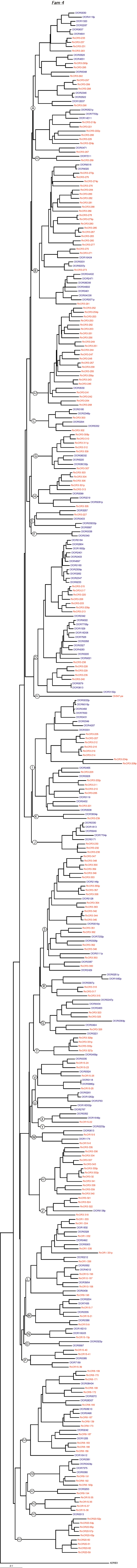

Supplement: Additional data file 6 — Phylogenetic tree for family 4. Dog and rat OR proteins belonging to the same family were aligned using ClustalW software [31] and a phylogram for each family was constructed using canine ADRB3 gene as the outgroup. Subfamilies are indicated by circled letters. Rat genes are shown in red and dog genes, in blue. [file gb-2005-6-10-r83-S6.jpeg]

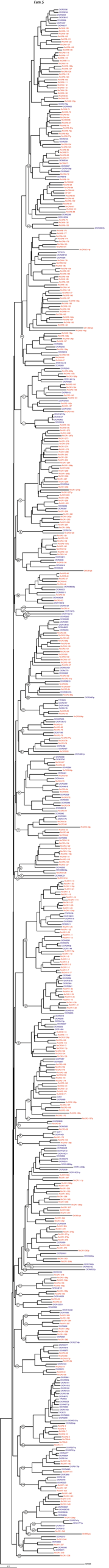

Supplement: Additional data file 7 — Phylogenetic tree for family 5. Dog and rat OR proteins belonging to the same family were aligned using ClustalW software [31] and a phylogram for each family was constructed using canine ADRB3 gene as the outgroup. Subfamilies are indicated by circled letters. Rat genes are shown in red and dog genes, in blue. [file gb-2005-6-10-r83-S7.jpeg]

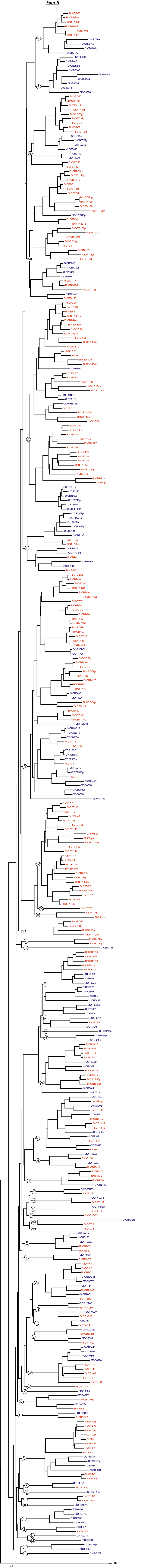

Supplement: Additional data file 8 — Phylogenetic tree for family 6. Dog and rat OR proteins belonging to the same family were aligned using ClustalW software [31] and a phylogram for each family was constructed using canine ADRB3 gene as the outgroup. Subfamilies are indicated by circled letters. Rat genes are shown in red and dog genes, in blue. [file gb-2005-6-10-r83-S8.jpeg]

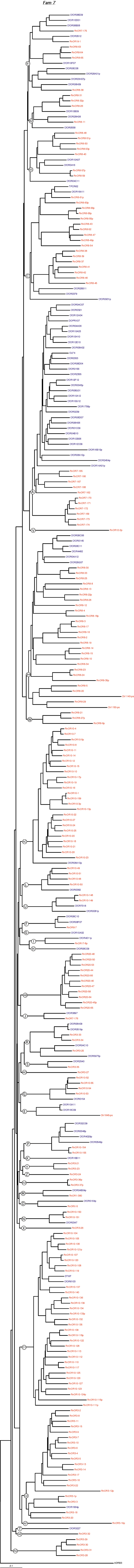

Supplement: Additional data file 9 — Phylogenetic tree for family 7. Dog and rat OR proteins belonging to the same family were aligned using ClustalW software [31] and a phylogram for each family was constructed using canine ADRB3 gene as the outgroup. Subfamilies are indicated by circled letters. Rat genes are shown in red and dog genes, in blue. [file gb-2005-6-10-r83-S9.jpeg]

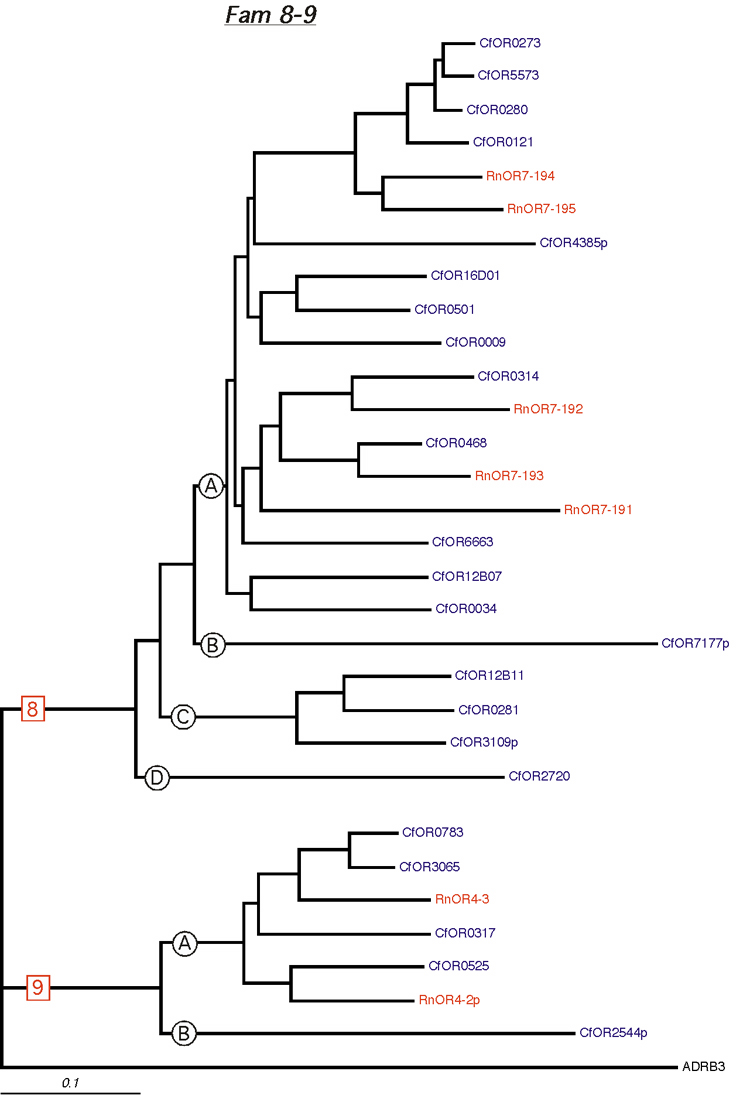

Supplement: Additional data file 10 — Phylogenetic tree for families 8-9. Dog and rat OR proteins belonging to the same family were aligned using ClustalW software [31] and a phylogram for each family was constructed using canine ADRB3 gene as the outgroup. Subfamilies are indicated by circled letters. Rat genes are shown in red and dog genes, in blue. [file gb-2005-6-10-r83-S10.jpeg]

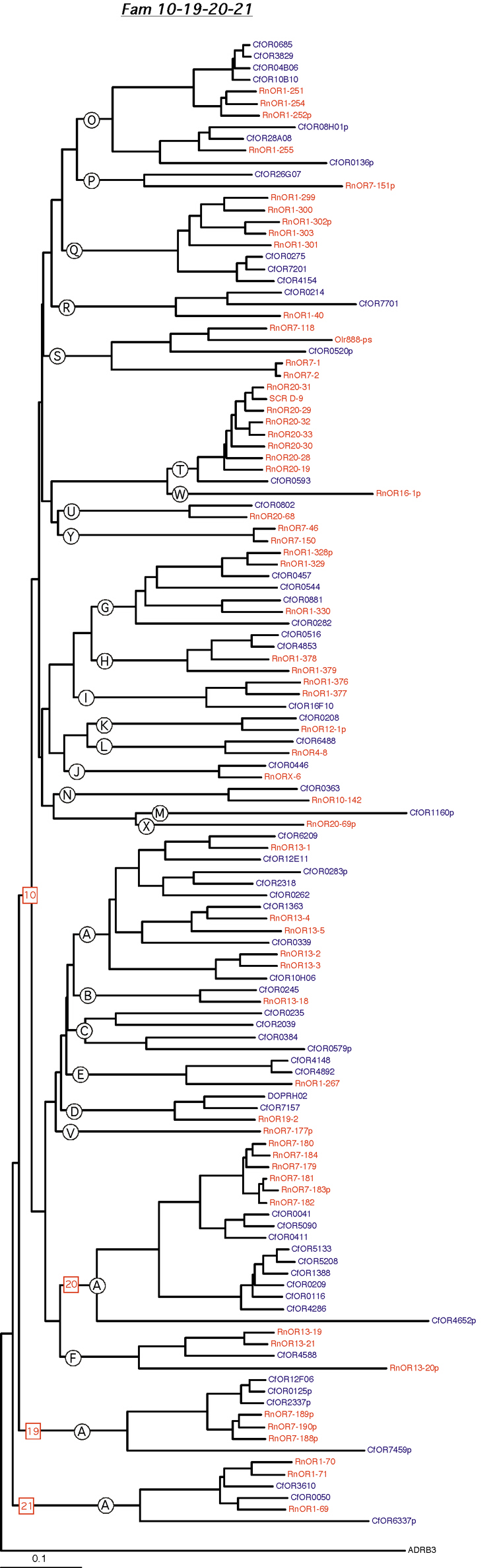

Supplement: Additional data file 11 — Phylogenetic tree for families 10-19-20-21. Dog and rat OR proteins belonging to the same family were aligned using ClustalW software [31] and a phylogram for each family was constructed using canine ADRB3 gene as the outgroup. Subfamilies are indicated by circled letters. Rat genes are shown in red and dog genes, in blue. [file gb-2005-6-10-r83-S11.jpeg]

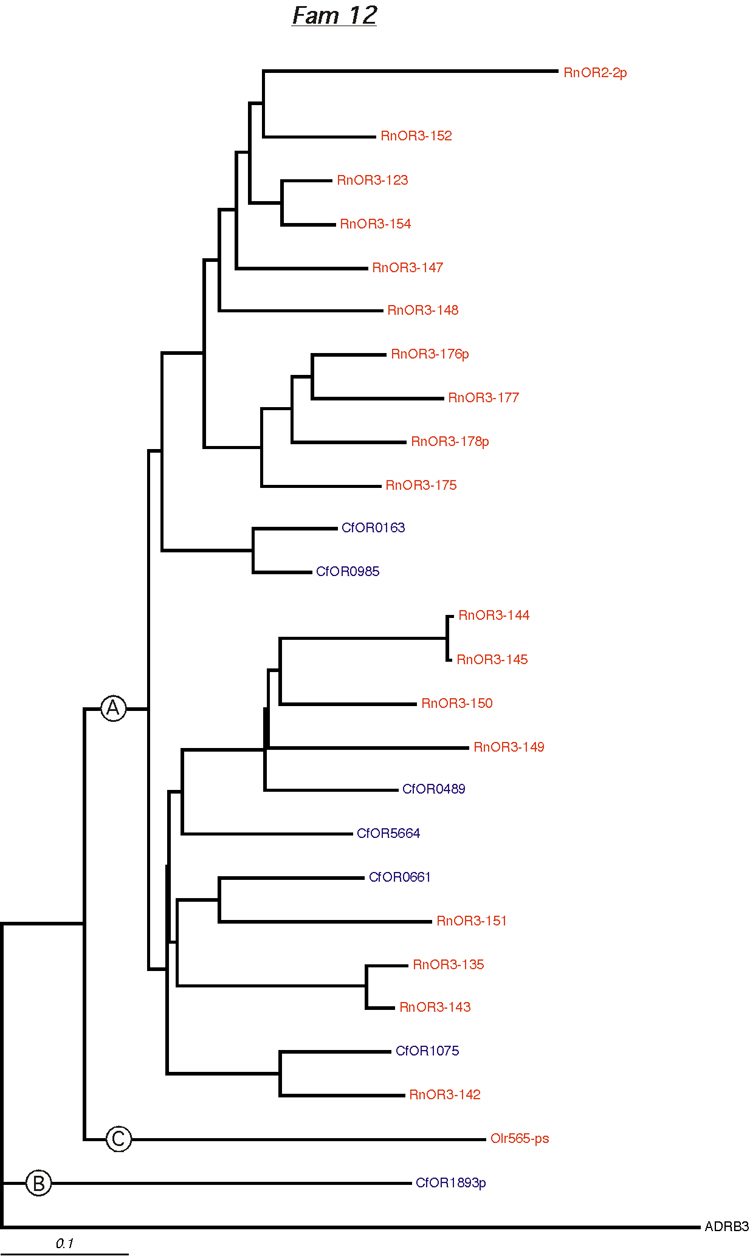

Supplement: Additional data file 12 — Phylogenetic tree for family 12. Dog and rat OR proteins belonging to the same family were aligned using ClustalW software [31] and a phylogram for each family was constructed using canine ADRB3 gene as the outgroup. Subfamilies are indicated by circled letters. Rat genes are shown in red and dog genes, in blue. [file gb-2005-6-10-r83-S12.jpeg]

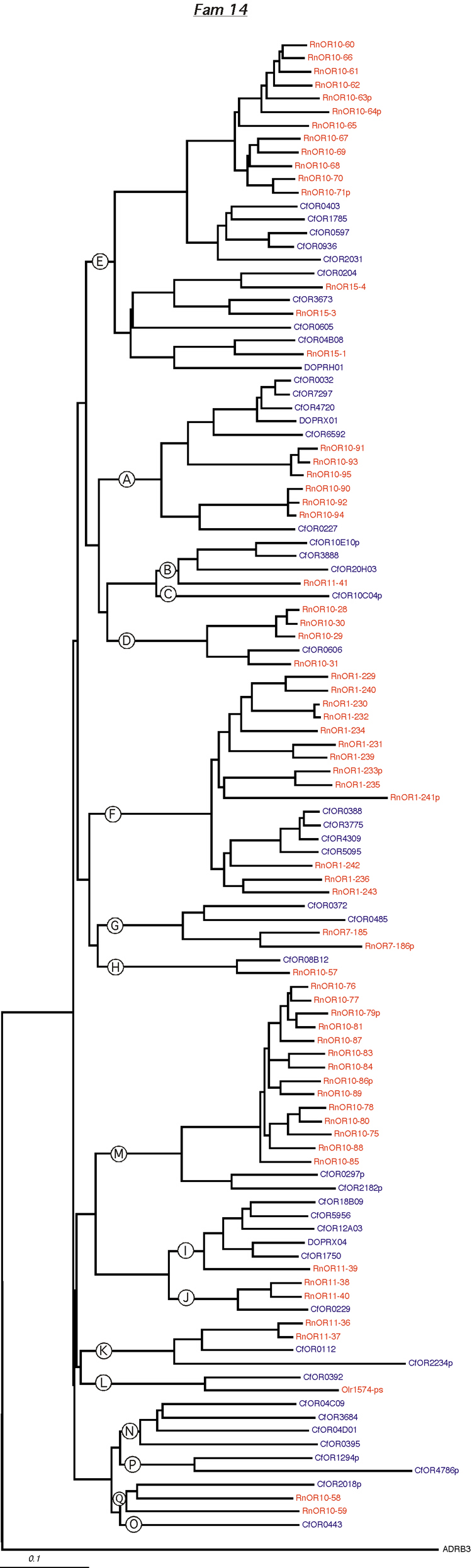

Supplement: Additional data file 13 — Phylogenetic tree for family 14. Dog and rat OR proteins belonging to the same family were aligned using ClustalW software [31] and a phylogram for each family was constructed using canine ADRB3 gene as the outgroup. Subfamilies are indicated by circled letters. Rat genes are shown in red and dog genes, in blue. [file gb-2005-6-10-r83-S13.jpeg]

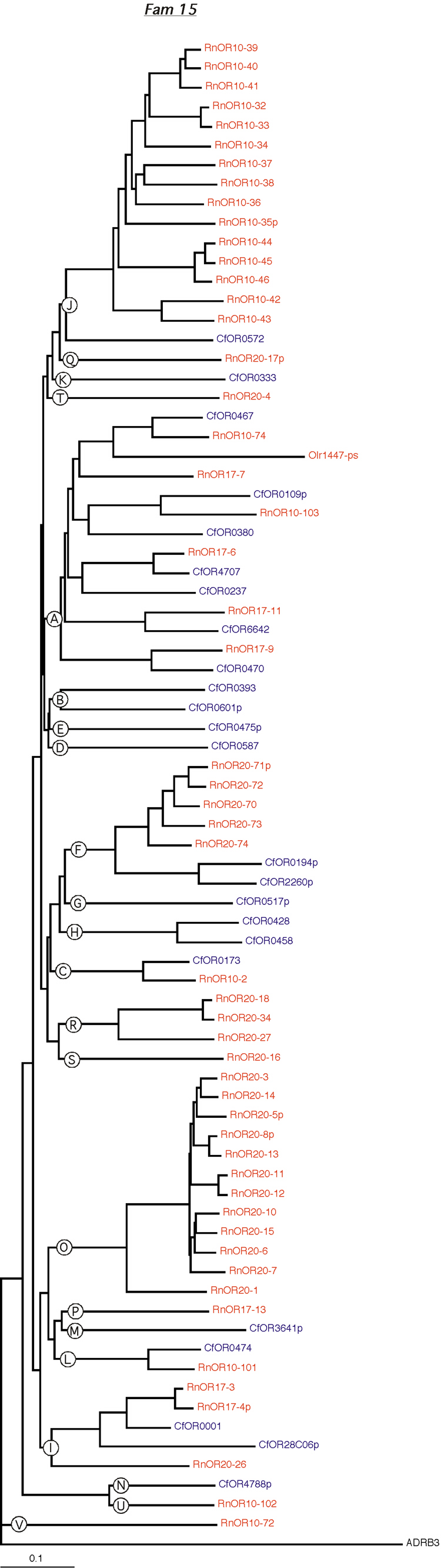

Supplement: Additional data file 14 — Phylogenetic tree for family 15. Dog and rat OR proteins belonging to the same family were aligned using ClustalW software [31] and a phylogram for each family was constructed using canine ADRB3 gene as the outgroup. Subfamilies are indicated by circled letters. Rat genes are shown in red and dog genes, in blue. [file gb-2005-6-10-r83-S14.jpeg]

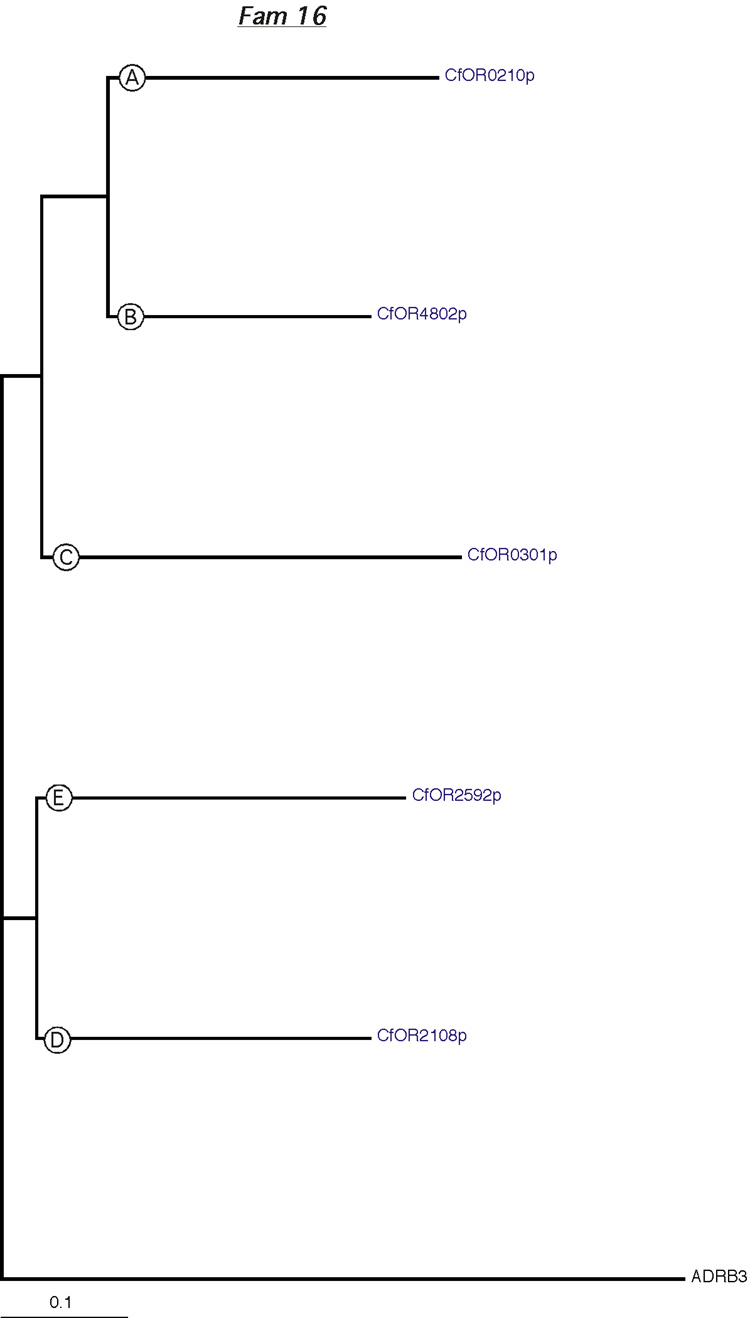

Supplement: Additional data file 15 — Phylogenetic tree for family 16. Dog and rat OR proteins belonging to the same family were aligned using ClustalW software [31] and a phylogram for each family was constructed using canine ADRB3 gene as the outgroup. Subfamilies are indicated by circled letters. Rat genes are shown in red and dog genes, in blue. [file gb-2005-6-10-r83-S15.jpeg]

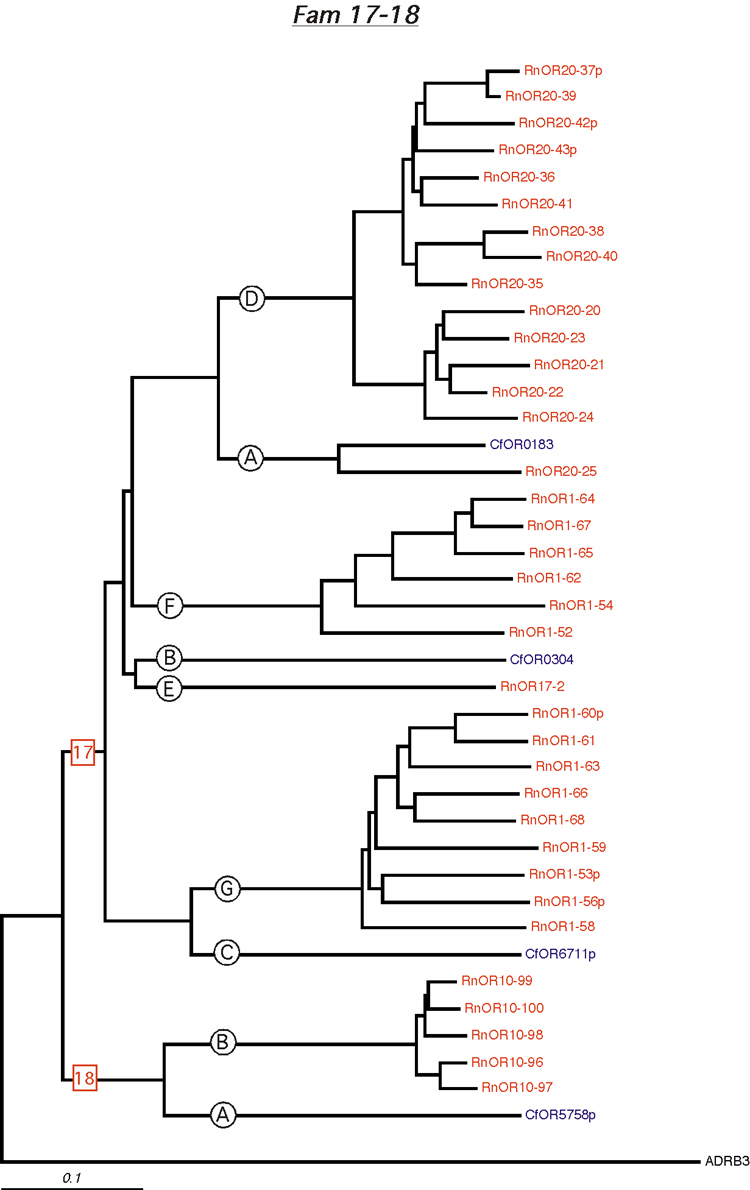

Supplement: Additional data file 16 — Phylogenetic tree for families 17-18. Dog and rat OR proteins belonging to the same family were aligned using ClustalW software [31] and a phylogram for each family was constructed using canine ADRB3 gene as the outgroup. Subfamilies are indicated by circled letters. Rat genes are shown in red and dog genes, in blue. [file gb-2005-6-10-r83-S16.jpeg]

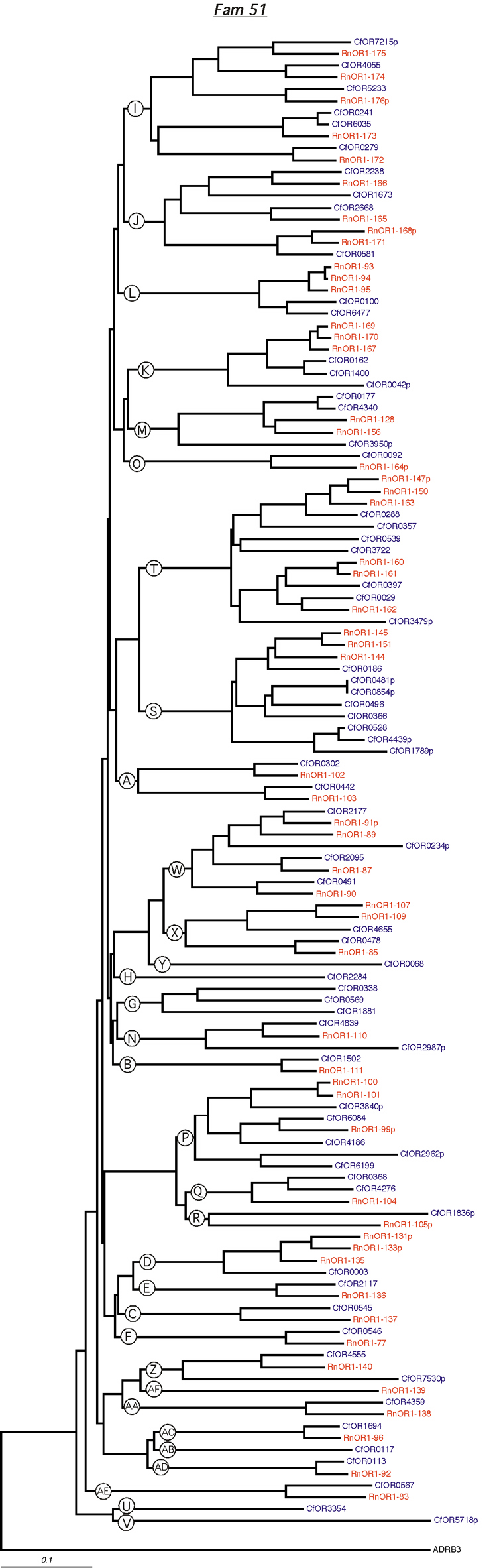

Supplement: Additional data file 17 — Phylogenetic tree for family 51. Dog and rat OR proteins belonging to the same family were aligned using ClustalW software [31] and a phylogram for each family was constructed using canine ADRB3 gene as the outgroup. Subfamilies are indicated by circled letters. Rat genes are shown in red and dog genes, in blue. [file gb-2005-6-10-r83-S17.jpeg]

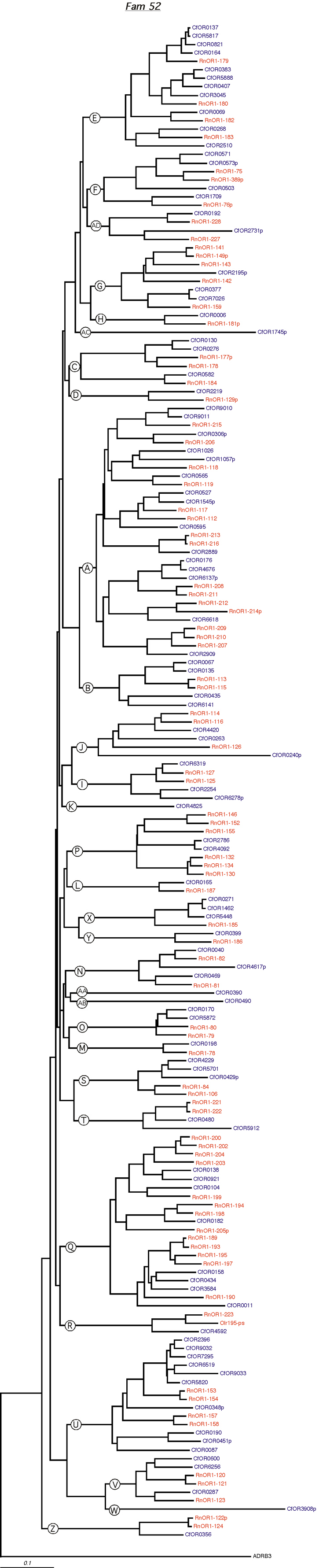

Supplement: Additional data file 18 — Phylogenetic tree for family 52. Dog and rat OR proteins belonging to the same family were aligned using ClustalW software [31] and a phylogram for each family was constructed using canine ADRB3 gene as the outgroup. Subfamilies are indicated by circled letters. Rat genes are shown in red and dog genes, in blue. [file gb-2005-6-10-r83-S18.jpeg]

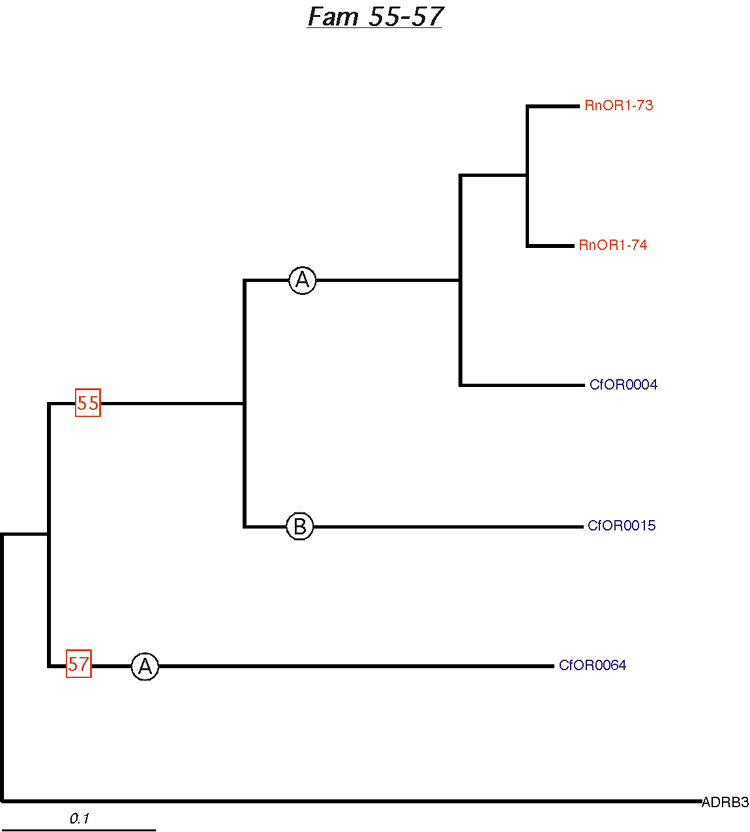

Supplement: Additional data file 19 — Phylogenetic tree for families 55-57. Dog and rat OR proteins belonging to the same family were aligned using ClustalW software [31] and a phylogram for each family was constructed using canine ADRB3 gene as the outgroup. Subfamilies are indicated by circled letters. Rat genes are shown in red and dog genes, in blue. [file gb-2005-6-10-r83-S19.jpeg]

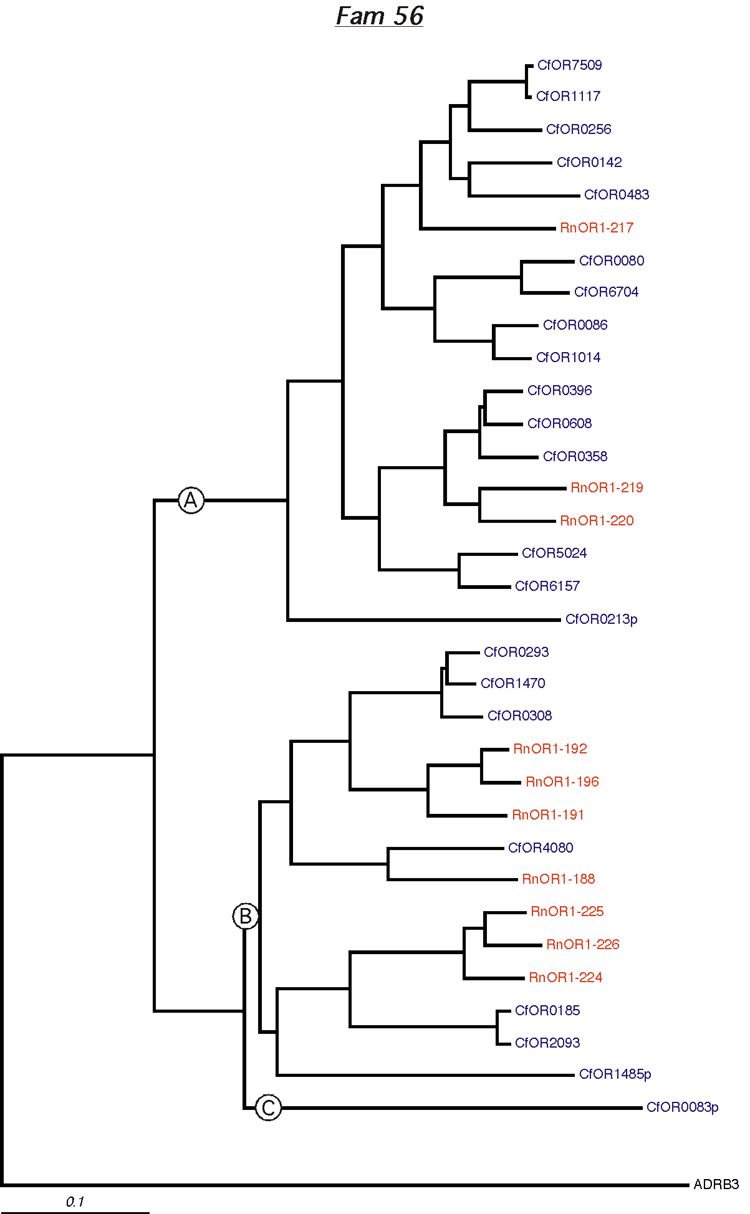

Supplement: Additional data file 20 — Phylogenetic tree for family 56. Dog and rat OR proteins belonging to the same family were aligned using ClustalW software [31] and a phylogram for each family was constructed using canine ADRB3 gene as the outgroup. Subfamilies are indicated by circled letters. Rat genes are shown in red and dog genes, in blue. [file gb-2005-6-10-r83-S20.jpeg]
